# Change of Cognitive Functions after Stroke with Rehabilitation Systems

**DOI:** 10.1515/tnsci-2019-0020

**Published:** 2019-05-06

**Authors:** Daiva Baltaduonienė, Raimondas Kubilius, Kristina Berškienė, Linas Vitkus, Daiva Petruševičienė

**Affiliations:** 1 Department of Rehabilitation, Medical Academy of Lithuanian University of Health Sciences, Kaunas, Lithuania; 2 Institute of Sports, Medical Academy of Lithuanian University of Health Sciences, Kaunas, Lithuania; 3 Republican hospital of Kaunas, Kunas, Lithuania

**Keywords:** stroke, cognitive training programme, virtual environment, occupational therapy

## Abstract

The objective of this study is to assess and compare the effect of applying a computerised cognitive training programme and virtual environment rehabilitation system on cognitive functions in patients after a stroke. Methods. A controlled trial included 121 persons referred to second stage rehabilitation. The subjects were differentiated into three impact groups by a single blinded trial. Results. The trial revealed that cognitive functions improved in all patient groups (p<0.001). A paired comparison analysis of all groups demonstrated a tendency for cognitive functions, evaluated by the MoCA–LT test, to be more strongly improved in patients who practised a computerised cognitive training programme during their OT sessions than those who did not (p=0.054). Conclusions. The final outcome of the trial was that cognitive functions significantly improved in patients who practised computerised cognitive training programmes or virtual environment rehabilitation systems, compared to those participants who only had occupational therapy sessions.

## 1 Introduction

Strokes are one of the main causes of disability in the Western world [1, 2]. Cognitive impairments of stroke patients [3] cover the range of 20 %–80 % and persist for 38 %–73 % of all cases [3, 4, 5]. The insufficient ability to concentrate upon the task, remember, learn, plan, use information, initiate and stop the activity, and solve problems is affected by cognitive function impairments. A stroke disturbs cognitive functions that include attention, memory, language, executive functions, spatial perception, and orientation.

Modern technologies for cognitive rehabilitation of stroke patients are becoming more accurate and effective [6]. Technological innovations allow computerised cognitive training (CCT) and the application of a virtual environment (VE) to render more cost-effective, acceptable, flexible, and multi-beneficial interventions [7, 8]. Increasing stroke rehabilitation with science–based computerised programmes and VE system practice is rapidly emerging [9].

Scientific papers have suggested that CCT may improve patients’ cognitive functions, especially working memory and motivation [10]. Computerised cognitive training programmes have a potential effect upon the recovery of cognitive functions and the training of particular cognitive functions [11, 12]. Scientists have identified that CCT programmes have a favourable effect upon language fluency and long–term as well as short–term memory [13], attention, working memory, and planning skills [14]. The application of computerised programmes and VE rehabilitation systems, positively affects cognitive function improvement and recovery in patients after stroke [7].

Other researchers have noted that conventional rehabilitation exercises may seem tedious due to their repetitive nature [15]. Moreover, a patient’s motivation is an important factor for rehabilitation success [15]. Computerised programmes and VE rehabilitation system training during rehabilitation sessions, enhance patient’s motivation, provide flexibility and reduce the treatment period [16]. Despite the impressive findings related to the morbidity of stroke consequences upon cognitive functions [17], the issue has still not received adequate observation [18], especially when combining VE sessions with CCT programmes. Nevertheless, there is modest evidence of VE advantages over conventional methods for the recovery of the cognitive functions after stoke [19]. The guidelines of the European Federation of Neurological Societies and American Heart Association/American Stroke Association do not exclude exact methods in occupational therapy for cognitive function improvement, and the evidence of it is still in discussion [17].

**Study objective**. To assess and compare the effect of applying a computerised cognitive training programme and virtual environment rehabilitation system on cognitive functions in persons with ischaemic stroke.

## 2 Materials and Methods

### 2.1 Participants and study design

**Figure 1:**
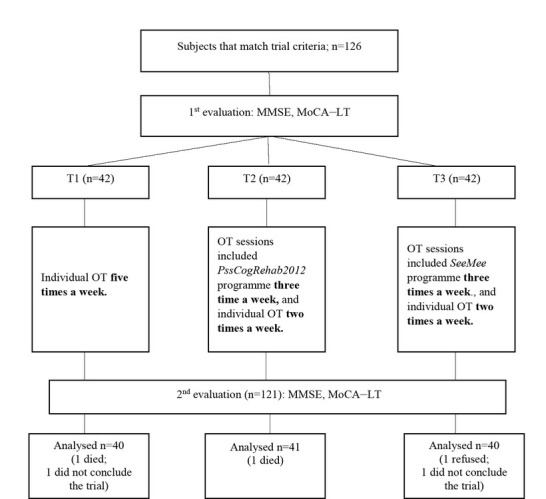
Trial organisation scheme

A randomised single blinded trial was performed in two Kaunas city hospitals after obtaining Kaunas Regional Biomedical Research Ethics Committee permission (No. BE–2–33). This study describes the outcome of the trial that was conducted in Lithuania and lasted for three years. The study objectives were introduced to trial participants and they voluntarily agreed to sign an informed consent form to participate. The inclusion criteria were as follows: subjects with first-time ischaemic stroke (10–14 days after the stroke), Barthel

index (BI) 50–65 points, Mini-Mental State Examination (MMSE) ≥11 points, subject’s agreement to participate in a trial, and vision and hearing suitable to evaluate cognitive functions and apply impact measures. Exclusion criteria involved BI from <50 or >65 points, MMSE <11 points, repeated stroke, subjects unable to speak or have diagnosed aphasia, neglect syndrome, and other neurological or mental health disorders. These criteria were chosen according to the law of the Ministry of Health of the Republic of Lithuania, and patients were treated in the Department of Physical Medicine and Rehabilitation for 32 days when they showed a BI of 50–65 points and results of the MMSE of 11–30 points. The trial involved 126 subjects with ischaemic stroke who were referred to rehabilitation. Patients were randomly assigned to study groups with a 1: 1: 1 allocation ratio, according to the rehabilitation registration journal. The first trial group (T1) consisted of 42 subjects, the second trial group (T2) involved 42 subjects, and the third trial group (T3) also consisted of 42 subjects. A total of 121 subjects established the trial, and 5 subjects left the trial because of the following reasons: 2 subjects died, 2 subjects discontinued the trial due to the impairment of their health condition, and one subject refused to participate in the trial (Figure 1).

Before and after the trial, cognitive functions of the subjects were evaluated with the MMSE, Montreal Cognitive Assessment test (original version 7.1) that was validated and adapted in Lithuania (MoCA–LT).

### 2.2 Intervention

The T1 group participated in a programme that included individual occupational therapy (OT) sessions (five times a week) with an aim of improving the existing cognitive impairments. During the sessions, participants were given tasks to train spatial perception, memory, attention concentration, and problem solving. All these functions were trained with the help of conventional “pencil–and–paper” cognition training tasks.

The T2 group participated in a programme that aimed to improve the existing cognitive impairments; this involved individual OT two times a week and individual OT sessions with a computerised cognitive training programme (*PssCogRehab 2012*, USA) three time a week. Individual OT included conventional “pencil–and–paper” cognition training tasks. Computer–based programmes that were practised three times a week included tasks from four modules (*Foundations I/ II, Memory I/ II, Problem Solving I/ II, Visuospatial I/ II*), and they were performed by a subject sitting in front of the computer screen. The tasks of these modules trained the subjects’ memory, problem solving, attention concentration, and spatial perception. The difficulty of the task was selected according to the subject’s cognitive function state.

The T3 group participated in a programme that provided individual OT two times a week, which was the same as the T1 group, and OT sessions included practised VE rehabilitation system activities (*SeeMe^ᴿ^ Brontes Processing*, Poland) three times per week. This system comprises a *Kinect* camera and specialised games for rehabilitation. Patients involved in VE rehabilitation sessions practised different game programmes and performed these programmes with hand movements sitting or standing 2–3 metres away from a motion sensor or camera. The following modules were applied: *Ball, React, Cleaner, Space, Warm up, Maze, Sorter*, and *Gym*. Programmes were selected depending on the participant’s cognitive function disorders.

All subjects participated in the 45-minute sessions five times a week. The trial lasted 32 days, and the hospitalisation lasted an average of 30.5 days. The first assessment of the subjects was performed during the first day of rehabilitation, and the second assessment was performed on the last day of rehabilitation. In addition, during the rehabilitation process, all subjects received physiotherapy, were counselled by a psychologist and social worker, and received pharmacological treatment.

### 2.3 Statistical analysis

Statistical data analysis was performed with IBM^®^ SPSS^®^ Statistics 22.0 for Windows software package. Qualitative data are expressed as the percentage, and quantitative data are expressed as the median (x_me_), minimum value (x_min_), maximum value (x_max_), and arithmetic mean $\left(\bar{x}\right)-{\text{x}}_{\text{me}}\left({\text{x}}_{\min,}{\text{x}}_{\mathrm{ma}x^{\prime }}\bar{x}\right).$For the comparison of two independent samples, the nonparametric Mann-Whitney test was used. For the comparison of two dependent samples, the nonparametric Wilcoxon test was used, and for the three samples, the nonparametric Kruskal– Wallis criteria was applied. The difference was considered statistically significant when p<0.05. The data were processed with the State Data Protection Inspectorate’s permission (No. 2R–1293 (2.6.1)).

## 3 Results

The study presents the results of the 121-subject trial, of which there were 75 (61.98 %) females and 46 (38.02 %) males. The average age of the subjects was 72.61 years (SD=10.79, range 46–90), the average age of the males was 70.17 (SD=11.60, range 46–89), and the average age of the females was 74.08 (SD=10.06, range 49–90). Before the trial, subjects were differentiated into homogenous groups according to gender, education, age, side of damage, and marital status (Table 1); there were no statistically significant differences established. Groups were homogeneous before the study prior to BI assessment. Prior to the treatment, there were no significant differences between groups for most characteristics and clinical evaluation variables. All subjects were right–handed.

**Table 1:** Demographic and descriptive parameters of the subjects

		T1 (n= 40)	T2 (n=41)	T3 (n=40)	Statistical criteria, p
	Age (mean ± SD)	74.33 ± 10.27	73.67 ± 10.10	69.71 ± 11.67	F(2, 112)=1.31; p=0.273
Education	Primary, n (%)	5 (12.5)	4 (9.76)	5 (12.5)	
	Secondary, n (%)	18 (44.0)	19 (46.34)	15 (37.5)	χ^2^(6)=4.281; p=0.639
	Specialised secondary, n (%)	5 (12.5)	10 (24.39)	11 (27.5)	
	Higher, n (%)	12 (30.0)	8 (19.51)	9 (22.5)	
Gender	Males, n (%) Females, n (%)	18 (45.0) 22 (55.0)	10 (24.4) 31 (75.6)	19 (47.5) 21 (52.5)	χ^2^(4)=5.505; p=0.064
Brain side injury	Right, n (%)	16 (40.0)	24 (58.5)	17 (42.5)	χ^2^(2)=3.301; p=0.192
	Left, n (%)	24 (60.0)	17 (4 1.5)	23 (57.5)	
Time to event	≤ 4 hours, n (%)	17 (42.5)	17 (41.5)	14 (35.0)	
(to stroke)	> 4 hours, n (%)	23 (57.5)	24 (58.5)	26 (65.0)	χ^2^(2)= 0.553; p=0.758
Marital status	Married, n (%)	20 (50.0)	19 (46.3)	26 (65.0)	
	Divorced, n (%)	6 (15.0)	9 (22.0)	1 (2.5)	χ^2^(6)=10.433; p=0.108
	Widow/Widower, n (%)	11 (27.5)	13 (31.7)	11 (27.5)	
	Single, n (%)	3 (7.5)	0	2 (5.0)	
	BI (mean ± SD)	54.63 ± 6.14	55.32 ± 6.13	53.63 ± 6.30	χ^2^(2)=5.891; p=0.053
	HADS-A (mean ± SD)	7.63 ± 3.39	6.98 ± 3.52	7.23 ± 3.61	χ^2^(2)=4.098; p=0.129
	HADS-D (mean ± SD)	6.95 ± 4.24	6.44 ± 3.81	6.28 ± 4.06	χ^2^(2)=0.404; p=0.817

T1 – the first trial group; T2 – the second trial group; T3 – the third trial group; BI – Barthel index; HADS-A – Hospital Anxiety and Depression Scale – anxiety subscale; HADS-D – Hospital Anxiety and Depression Scale – depression subscale.

The study findings (Table 2) indicated that subjects’ cognitive functions significantly improved (p<0.001) in all three trial groups. Separate group analysis of the MMSE test results after the trial showed the difference between the findings of all three trial groups (χ^2^(2)=8.916; p<0.05). Paired comparison analysis of all groups revealed significant result differences between the T1 and T2 groups (p=0.026), and between the T1 and T3 groups (p=0.033). Overall, it was identified that cognitive functions were significantly improved in participants who had OT sessions involving the CCT programme or VE rehabilitation system.

**Table 2:** MMSE and MoCA–LT test results of the trial groups before and after the trial

	Variable		Time 1			Time 2		Z; p

		Median	Mean	SD	Median	Mean	SD	
T1	MMSE	23.50	22.95	3.52	26.00	25.13	3.13	-4.436; p<0.001
	MoCA-LT	19.00	18.08	4.49	21.50	21.03	4.85	-4.121; p<0.001
T2	MMSE	23.00	23.44	3.78	28.00	26.93	2.41	-5.286; p<0.001
	MoCA-LT	18.00	17.78	3.82	24.00	23.68	3.70	-5.588; p<0.001
T3	MMSE	25.00	24.88	2.58	27.00	26.90	2.56	-5.169; p<0.001
	MoCA-LT	19.50	19.93	4.04	24.00	23.25	3.92	-4.892; p<0.001
χ^2^, p	MMSE		χ^2^(2) = 6.915; p = 0.032			χ^2^(2) = 8.916; p = 0.012		
	MoCA-LT		χ^2^(2) = 5.139; p = 0.077			χ^2^(2) = 6.555; p = 0.038		

MMSE – Mini-Mental State Examination; MoCA-LT – Montreal Cognitive Assessment Test validated and adapted in Lithuania; T1 – the first trial group; T2 – the second trial group; T3 – the third trial group.

The analysis of the MoCA–LT test results (Table 2) revealed that after the trial in both T2 and T3 groups, the cognitive function value reached 24 points. These results indicated a statistically significant improvement in cognitive functions in all groups (p<0.001). The inter–comparison of the group evaluation results before the trial revealed no difference between the groups regarding the MoCA–LT test findings (χ^2^(2)=5.139; p=0.077). However, after the trial, MoCA–LT test findings showed differences between all three trial groups (χ^2^(2)=6.555; p<0.05). Paired comparison analysis of all groups showed a tendency towards the improvement of cognitive functions being stronger in patients who practised the CCT programme during the OT sessions (p=0.054).

**Figure 2:**
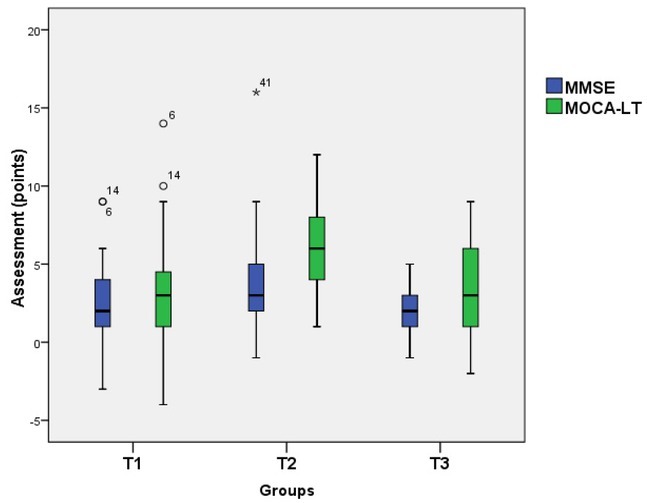
Changes of MMSE and MoCa-LT test results (T1 – the first trial group; T2 – the second trial group; T3 –the third trial group)

The comparison (Figure 2) of the abovementioned changes between the groups demonstrated that the changes reflected in the MMSE test results were different in all three trial groups (χ^2^(2)=7.834; p<0.05). Paired comparison analysis of all groups showed a significant difference only between the T1 and T2 groups (p=0.038). Analysis of the cognitive function changes reflected in the MoCA–LT test results revealed that, on average, cognitive functions in the T1 group improved by 3.10±3.60 points. In the T2 group, it improved by 5.90±2.53 points, and in the T3 group, it improved by 3.33±3.01 points. The analysis of the changes in all groups reflected in the MoCA–LT data, demonstrated that the results were different in all three trial groups (χ^2^(2)=20.142; p<0.001). Paired comparison analysis of all groups showed a significant difference in cognitive function changes between the T1 and T2 groups (p<0.001).

## 4 Discussion

The choices of science–based interventions related to the rehabilitation of cognitive functions after stroke are increasing. However, reviews in the scientific literature of trials combining the impact of two cognitive function training programmes (computer–based cognitive training and VE rehabilitation systems) are infrequent during the early stage of rehabilitation after stroke.

The results of this study revealed benefits of CCT programmes and VE systems in the process of rehabilitation. Park J.H. et al. (2015) practised a computerised programme *CoTras* and identified that the CCT programme greatly improved patients’ cognitive functions during the early stage of rehabilitation after stroke [20]. The study performed by Zucchella Ch. et al. (2014) demonstrated considerable cognitive function changes in a group that used CCT programmes. The analysis of the particular group results of the latter study showed significant changes in the fields of memory and spatial attention [21]. The above–mentioned findings are supported by systemic reviews performed by other scientists who suggested that computer–based cognitive rehabilitation is an effective cognitive function training tool for stroke patients [12, 22, 23, 24]. The findings of the present study revealed significant positive changes in the cognitive functions in the trial groups assessed before and after the trial (p<0.001); however, paired comparison analysis of the groups after the trial showed significant differences between the T1 and T2 groups (p<0.05), and between the T1 and T3 groups (p<0.05) reflected in MMSE test results. After the impact measures were applied, the data of the cognitive function evaluation reflected in the MoCa–LT test results highlighted the difference between the trial groups (p<0.05). Detailed analysis and paired comparison of all groups demonstrated a tendency where cognitive functions were more strongly improved in patients who practised the CCT programme during the OT sessions than those that did not (p=0.054). Scientific systemic review (2015) indicates that computer-based cognitive training as well as VE rehabilitation systems were moderately effective in affecting the long-term cognition improvement in patients with high risk of cognitive function impairment manifestation [7]. Nevertheless, due to high variability of the studies, it becomes difficult to determine whether the computer-based programme or the VE rehabilitation system produced a more significant effect upon the improvement of cognition in comparison with conventional means [7].

To identify the state of the cognitive functions, the studies employed numerous validated tools, such as MMSE, Neuropsychological Test Batteries, MoCA, Loewenstein Occupational Therapy Cognitive Assessment, and others [12]. The present study applied two tools: MMSE and MoCA–LT. The comparison of the means of the abovementioned test findings before and after the trial, revealed a stronger change in the means of the group that practised the CCT programme than those that did not (p<0.05). Thus, it can be assumed that practising the CCT programme had a stronger impact on cognitive function improvement than not using it. However, it would be expedient to perform further studies analysing the changes of particular cognitive functions.

Recommendations for cognitive rehabilitation are rather general and usually differ at a low evidence–based level [17]. Most studies that analyse the applicability of CCT programmes or VE rehabilitation systems concentrate on the training of specific cognitive fields such as memory [23, 24, 25, 26], attention concentration [23, 25, 27], or other functions [24]. The present study focused on assessment of changes in overall cognitive functions.

A large number of trials apply cognition training interventions at a later stage after stroke treatment; however, as some experts have stated, the recovery of cognitive functions is faster when the interactive rehabilitation programmes are practised during the acute or subacute stroke stage [12, 20, 21]. Our research showed the importance of applied interactive approaches during the early stage of rehabilitation, highlighting the significant improvement of cognitive functions in all trial groups.

As the scientific literature suggests, there is a wide variety of CCT programmes and VE rehabilitation systems aimed at training cognitive functions and offering different levels of duration and intensity [7, 12]. In the present trial, the interactive rehabilitation means were practised for four weeks; meanwhile, the duration in other trials varied from two weeks to three months or longer [12, 20, 21, 22, 23, 24, 28, 29]. Researchers indicated the need for an appropriate practise duration for the training to be more effective, since a short-termed cognitive training usually renders only a brief and temporary effect [10, 12]. However, van de Ven et al. indicated that the connection between the duration of training and the effectiveness of the impact remained unclear [30]. The interactive experience should be perceived by the OT specialist as well as by the patient as a very positive factor allowing continued treatment without fatigue or boredom [31, 32].

American Stroke Association recommendations state the expedience of an enriched environment to increase the subject’s engagement in cognitive activities (evidence level A) [17]. Therefore, the recommendation is use of CCT programmes and VE tasks, and allowing access to the internet for the enrichment of a stroke patient’s environment during rehabilitation to encourage these patients to engage more actively in the proposed activities, and in this way, improve their cognitive functions. The use of CCT programmes during the OT sessions allows meeting real patient needs; it also increases their motivation since the tasks are individualised and enables monitoring of the patient’s performance for evaluation and feedback [33]. When compared with conventional OT sessions, the engagement of patients in interactive activities is stronger, they strive to complete the task, seek new challenges, and train their imagination; these factors were also observed in the present study. In their study, Lee et al. indicated that the use of CCT programmes improved the achievements of the trial subjects, and the patients were proud to be able to use the computer and show it to others [34]. The subjects of the present trial were also highly interested in the activities that were practised; they provided more comments on the success and failure, and expressed their emotions. Thus, it is possible to consider that the positive atmosphere made an impact on improvement of the results. These considerations are supported by previous studies from different experts [33, 35].

Other researchers have stated that CCT programmes as well as VE rehabilitation systems might be very beneficial if practised in combination [28, 29, 36] rather than separately, although both of these tools have an advantage over the conventional cognition stimulation means [29]. In the present trial, each group practised a separate interactive tool, and their impacts were not combined. However, in future trials, there is an intention to combine the application of these means.

## 5 Limitation of the study

The generalisation of the study results highlight several limitations. First, we would recommend the analysis of a general change in cognitive functions in a detailed evaluation of particular cognitive functions. In addition, we would recommend successive monitoring of the impact of the practised CCT programmes and VE rehabilitation system for the assessment of a persistent benefit. At the beginning of the trial, the elderly intended to refuse to participate and indicated that they did not know how to operate the interactive means, and they were stressed because of lack of self–confidence due to a possible inability to perform the given tasks properly. Another limitation of this study was that we had to overestimate the limits of self-sufficiency (BI 50-65) and cannot say what the adaptability of this data would be in another patient’s self-sufficiency level. Therefore, it would be appropriate to design a simpler operation for the applied technologies.

## 6 Conclusions

Cognitive functions after rehabilitation have improved for all groups. Better estimates of cognitive functions were a computerised cognitive training programme or virtual environment rehabilitation system practised during OT sessions significantly improved the cognitive functions of patients with ischaemic stroke than in a group of people who were only in occupational therapy sessions. Applying innovative rehabilitation measures during OT sessions can assist patients in achieving better outcomes as well as restoring, stabilising, and improving cognitive functions during the early stage of rehabilitation after an ischaemic stroke.
